# Despite WT1 binding sites in the promoter region of human and mouse nucleoporin glycoprotein 210, WT1 does not influence expression of GP210

**DOI:** 10.1186/1477-5751-3-7

**Published:** 2004-12-21

**Authors:** Magnus Olsson, Milton A English, Jacqueline Mason, Jonathan D Licht, Peter Ekblom

**Affiliations:** 1 Department of Cell and Molecular Biology, Section for Cell and Developmental Biology, Lund University, Sweden; 2 Mount Sinai School of Medicine, 1425 Madison Avenue, New York, NY 10029, USA

## Abstract

**Background:**

Glycoprotein 210 (GP210) is a transmembrane component of the nuclear pore complex of metazoans, with a short carboxyterminus protruding towards the cytoplasm. Its function is unknown, but it is considered to be a major structural component of metazoan nuclear pores. Yet, our previous findings showed pronounced differences in expression levels in embryonic mouse tissues and cell lines. In order to identify factors regulating GP210, the genomic organization of human GP210 was analyzed *in silico*.

**Results:**

The human gene was mapped to chromosome 3 and consists of 40 exons spread over 102 kb. The deduced 1887 amino acid showed a high degree of alignment homology to previously reported orthologues. Experimentally we defined two transcription initiation sites, 18 and 29 bp upstream of the ATG start codon. The promoter region is characterized by a CpG island and several consensus binding motifs for gene regulatory transcription factors, including clustered sites associated with Sp1 and the Wilms' tumor suppressor gene zinc finger protein (WT1). In addition, distal to the translation start we found a (GT)n repetitive sequence, an element known for its ability to bind WT1. Homologies for these motifs could be identified in the corresponding mouse genomic region. However, experimental tetracycline dependent induction of WT1 in SAOS osteosarcoma cells did not influence GP210 transcription.

**Conclusion:**

Although mouse GP210 was identified as an early response gene during induced metanephric kidney development, and WT1 binding sites were identified in the promoter region of the human GP210 gene, experimental modulation of WT1 expression did not influence expression of GP210. Therefore, WT1 is probably not regulating GP210 expression. Instead, we suggest that the identified Sp binding sites are involved.

## Introduction

Nuclear pore complexes (NPCs) provide the only known gateway for transport of RNAs to the cytoplasm and bidirectional transport of proteins between the nucleus and the cytoplasm. The NPC in vertebrates has an estimated mass of approximately 125 Mda. Structural studies suggest an octagonal rotational symmetry framework, from which 50–100-nm long fibrils extend into the nucleoplasm and cytoplasm. A comprehensive inventory of all NPC constituents has been made for yeast [1] and metazoans [2]. A polypeptide profile from purified rat liver NPCs revealed ~50 putative nucleoporins [3].

In the list of metazoan nucleoporins, there are only two integral membrane proteins, gp210 [4-6] and POM121 [7,8]. Both have been localized to the NPC structure, each with a distinct membrane topology and amino acid motifs. Primarily due to their location, both proteins are presumed to anchor NPCs by the nuclear envelope and to assemble nucleoporins postmitotically. No binding partners have so far been identified for either of these proteins. The 121-kDa pore membrane protein POM121 [7,8] is located in the pore membrane domain of the NPC with a short (29 residues) N-terminal tail protruding into the lumen of the nuclear envelope, with the C-terminus facing the cytoplasm [8]. POM121 contains a C-terminal tandem sequence repeat of a core XFXFG motif interrupted by hydrophilic spacers. These motifs typical for nucleoporins and have been shown to interact with components of the soluble transport machinery [3,9].

In contrast to POM121, gp210 has an inverted topology with its main bulk residing in the lumen of the NE and only a short 58 residue C-terminal portion facing the NPC [5,6]. The amino acid-sequence of gp210 lacks pentapeptide repeats indicating no direct interaction with the mobile receptors directing nucleocytoplasmic transport [5,10]. A 23-amino-acid hydrophobic peptide residing in the luminal part of gp210 has been predicted to be involved in formation of new pores acting as a nuclear membrane fusion agent [5,11]. It has also been experimentally shown that the C-terminus of gp210 is involved in nuclear pore dilation [11], even though this is not a conserved sequence in different species [12]. Remarkably, it has also been shown that gp210 is essential for viability of human HeLa cells and C. Elegans [13]. A fraction of the cellular pool of gp210 can form dimers that may constitute a lumenal submembranous protein skeleton [14].

The primary sequence of gp210 is known for rat [5] and mouse [10]. Interestingly, whereas several nucleoporins found in vertebrates have homologues in the completed yeast genome, no such similarities have so far been detected for POM121 or gp210. Possibly, this could be related to the fact that the yeast nuclear membrane does not break down during cell division, and assembly regulators are not needed. In a comprehensive analysis of a highly enriched NPC fraction, presumably containing all yeast NPC proteins [1], only three transmembrane nucleoporins were detected, but these have no resemblance with gp210 or POM121. Thus, if the role of POM121 or gp210 in metazoans is to anchor the NPC, different proteins or mechanisms should be involved in the anchorage of yeast NPCs. Mouse gp210 was initially identified as an early response gene to induction of metanephric kidney development and data from other embryonic tissues confirmed the differential distribution of its mRNA [10] and protein [15]. This suggested a novel cell-type specific regulation of gp210. It was thus of interest to characterize the promoter region of the human GP210 gene.

In the current study we present the genomic structure of the human integral membrane glycoprotein 210 gene (GP210), the open reading frame sequence and a promoter region analysis. This was done in silico by taking advantage of the available human genomic sequence. Transcription start sites were determined experimentally by RNA ligase mediated rapid amplification of cDNA ends. Computer-assisted searches of the promoter sequence indicated putative consensus binding sites for transcription factors involved in tissue specific gene regulation. We also identified of shared putative *cis*-acting elements in the human promoter and its mouse counterpart. Several putative Wilms tumor suppressor binding 1 sites were found. Nevertheless, experimental overexpression of WT1 in SAOS osteosarcoma cells did not influence GP210 mRNA expression.

## Results

### Organization of the human GP210 gene

**Figure 1:**
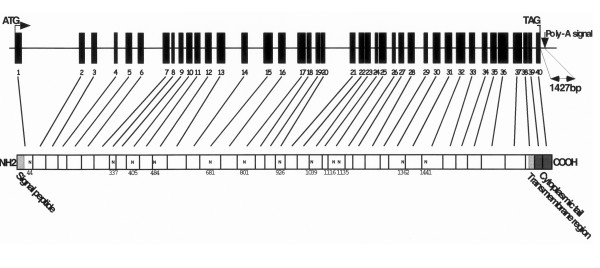
**Genomic organization of GP210 and a model of the deduced amino acid chain of GP210**. Exons (black boxes) and intron sizes are scaled individually. In silico predictions of a signal peptide, the transmembrane region and 12 putative N- linked glycosylation sites (N).

We initially assumed that mouse gpP210 was a member of a yet undiscovered large family of tissue-specific nuclear pore membrane proteins, and initially named it POM210 [10] to emphasize the similar subcellular distribution with POM121 [7]. Since current data suggest a surprisingly low amount of pore membrane proteins both in vertebrates and yeast, renaming is unnecessary. A BLAST homology search was performed using the mouse gp210 cDNA sequence (POM210, accession AF113751) against the working draft sequences of the human genome. This identified a completed contig-component (clone RP11-220D14, accession AC090942.1) localized to chromosome 3, in a region defined by three genomic markers (stSG4499, Cda14e10 and WI-9637). These markers were cytogenetically positioned to 3p25.1. By comparing to the mouse cDNA sequence and taking advantage of the exon/intron prediction program provided by the Genscan web server, 40 exons covering 102551 bp were defined (see Table 1, additional file 1). The exons ranged between 63 (exon 24) and 251 (exon 36) bp. All exon-intron junctions conformed to the consensus splice donor (GT) and acceptor (AG) sites, except for the splice donor sites of intron 7 (AT) and10 (GC). The introns sizes were between 74 (intron 38) and 20198 bp (intron 1). Introns were classified relative to codon interruption, as follows: phase 0 (no codon interruption), phase 1 (interruption between first and second base) and phase 2 (after second base). Exons were interrupted by introns of all phases. Most introns were of phase 0 (55%). A number of efforts where made to identify alternatively spliced products using PCR with primer pairs directed to high probability putative splice variant exons. However, no such variants could be found. Manually, we could identify one single polyadenylation signal (AATAAA) 1423 nucleotides downstream of the translation stop codon (Fig. 1). The exons formed an ORF of 5664 bp including the stop codon.

### Primary structure of GP210

The deduced amino acid sequence of human GP210 contains 1887 residues, predicting a molecular mass of 205 198 Da and a pI of 6,41 of the non-processed protein. Alignment to the corresponding mouse and rat sequences displayed a high degree of homology (91,8% similarity and 88,9% identity compared to the primary structure of the rat protein). One insertion, an alanine at position 1858, makes the GP210 one residue longer compared to rat and mouse GP210. A signal peptide cleavage consensus site could be defined between residues 25 and 26 using the SignalP algorithm (Fig. 1). This signal sequence shows no resemblance to previously reported GP210 sequences. Hydrophobicity values along the deduced amino acid chain identified several putative membrane spanning regions. One of these (residues 1809 to 1828) corresponded to a domain mapped in the rat orthologe [5], leaving 59 residues facing the nuclear pore. Motifs found scanning the sequence through the ExPASy-Prosite database included 12 potential N-glycosylation sites (outlined in Fig. 1), and numerous putative consensus sites for various kinase related phosphorylations. Two cAMP- and cGMP dependent protein kinase sites (residues 1089, 1874), two tyrosine kinase sites (residues 227, 922), 30 protein kinase C phosphorylation sites and 26 casein kinase II phosphorylation sites were found. The sites associated to PKC and CK2 phosphorylation were evenly distributed throughout the sequence. Blast homology searches revealed a vast number of EST clones containing GP210 sequence and an 871 amino acid partial sequence of a hypothetical human protein KIAA09906 (accession Xp051621) identical to the C-terminal end of the full length translated GP210 open reading frame.

### Identification of the transcription start

**Figure 2:**
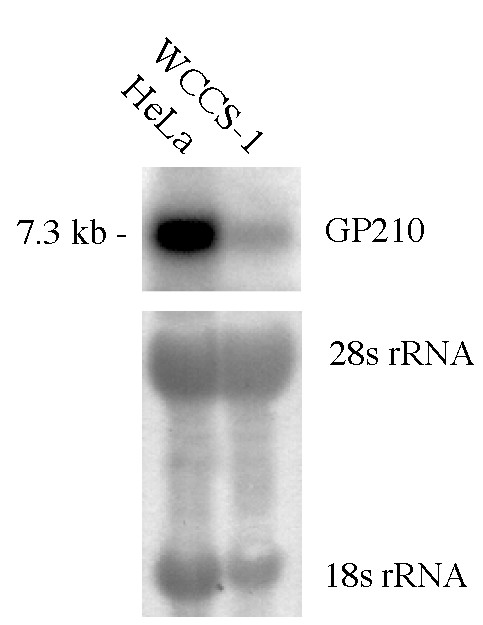
**Northern blot analysis of HeLa cell and WCCS-1 mRNA**. A 935 bp GP210 specific cDNA probe was hybridized to 10 μg of HeLa and WCCS-1. Loading and RNA quality was controlled by a methyleneblue staining of 18 and 28s rRNA.

**Figure 3:**
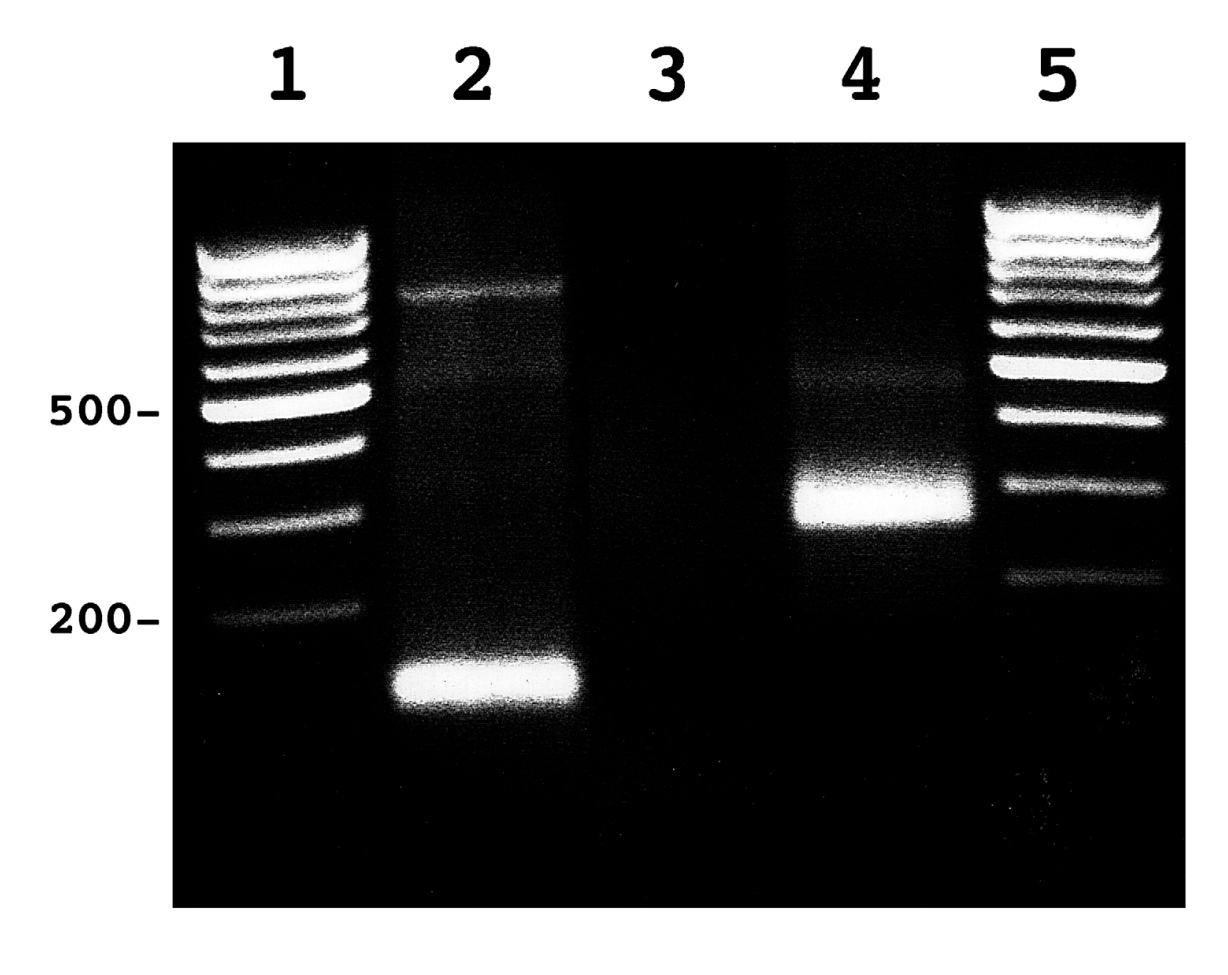
**Determination of 5 prime end of GP210 mRNA**. Transcription start site were determined analyzing nested PCR products generated with gene (fig. 5) and adapter specific primers. Lane 1, Marker. Lane 2, Nested PCR product obtained using two primer pairs specific to sequences within the adapter and the first exon. Lane 3, Negative water control. Lane 4, Positive control using bacterial adapter ligated cDNA and specific primers. Lane 5, Marker.

**Figure 4:**
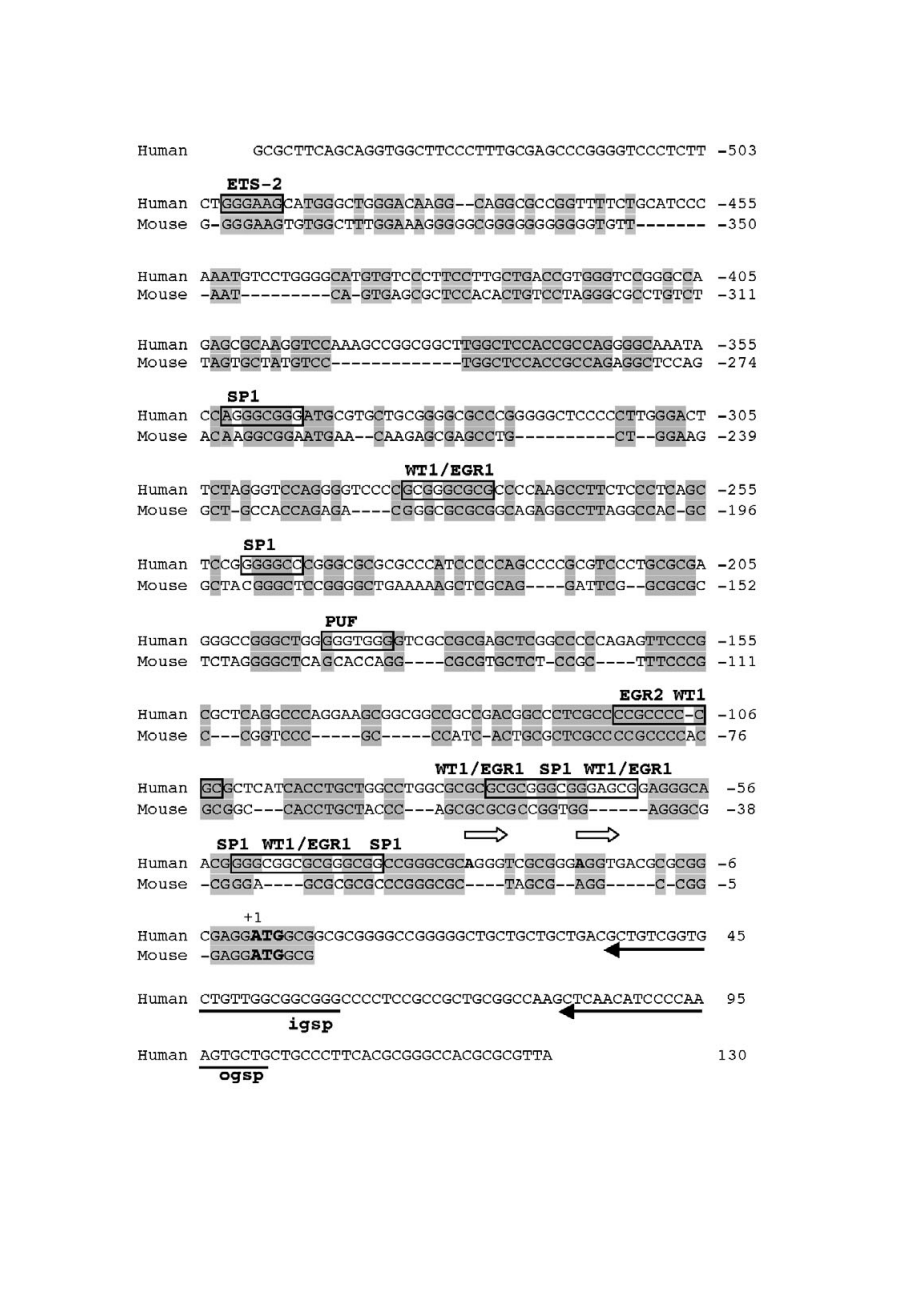
**Promoter analysis human GP210 and homology to its mouse counterpart**. A 500 bp sequence upstream of translation start site was analyzed for the presence of consensus transcription factor binding sites. Upper lane is the human sequence, and lower sequence is mouse. Homology in the (GT)n repeat between human and mouse genomic sequences and its position relative to the translation start codon. The translation start site is in bold and numbered +1. Homology to the mouse sequence is marked in grey, Outer gene specific primer (ogsp) and inner gene specific primer (igsp) used for transcription start definition using RLM-RACE are underlined with arrows. The transcription initiation sites are positioned with empty arrows and the start nucleotide is in bold. Putative transcription binding motifs are underlined. Some elements for different transcription factors overlap. Only sense strand binding sites were considered. Legend: Sp1, Simian-virus-40-protein 1; EGR2, Early growth response gene 2; WT1, Wilms' tumor zink finger protein 1; PuF, c-myc purine-binding transcription factor.

The sequence 1 kb upstream of the ATG start codon possesses neither TATA or CAAT boxes, but contains scattered initiator (Inr) elements (consensus Py Py C A N T/A Py Py) [16]. In order to determine the transcription start site (tss), we therefore performed RNA ligase mediated rapid amplification of cDNA ends [17]. By Northern blotting using a 935 bp cDNA probe (nt 4286–5220), a single transcript of 7,3 kb was seen in two different tumor cell lines (Fig 2). The size of the mRNA corresponds to the sum of the open reading frame and 3'-UTR including a potential poly A tail. Since expression was much more abundant in HeLa cells than in Wilms tumor cells, we used HeLa cell total RNA as a template. The nested PCR (see material and methods) gave a major specific cDNA product of approximately 80 nucleotides (fig. 3, lane 2). Sequence analysis of 10 independently ligated PCR products obtained using nested adapter- and gene specific primers (outlined in fig. 3) revealed two different amplified, GP210 specific fragments of similar length, indicating two alternative tss (Fig. 4). Out of ten clones sequenced, eight ended at position -29 and two at position -18 upstream ATG. In an identical analysis using poly-A+ RNA from a human fetal myoblast cell line G6, 7 clones ended at position -29 and 3 at position -18 upstream ATG. These findings argue for a major tss at -29. In addition, to confirm our findings we performed sequence homology searches in the human EST clone database at NCBI and elsewhere. The results revealed no reported cDNA sequences located upstream of the experimentally determined start site.

### Promoter sequence analysis

Analysis of the sequence surrounding the translation start site with the GRAIL program predicted a 1236 bp long CpG island, with a GC content of 75.3% starting 434 bp upstream of ATG, covering the first exon and extending into the first intron. We used a variety of promoter and transcription factor binding site algorithms to analyse the region upstream the start site for GP210, including TESS and Matinspector. By selecting for perfect matching and human consensus motifs a restricted number of putative transcription factor binding sites were found. Using these criteria, 22 motifs recognized by 14 different factors were defined on the sense strand, evenly distributed within 1000 bp proximal to the translation start codon (See Table 2, additional file 2). Seven Sp1 binding motifs were identified [18], five of them clustered in a region spanning 315 bases, starting eight nucleotides upstream of the major tss (Fig. 4). Four putative binding motifs for EGR1/WT1 (consensus GXGXGGGXG) were mapped within the same promoter region, starting at positions -47, -70, -76 and -283. Two of these matched completely with this consensus sequence, whereas two contained one mismatch in the 9bp-binding motif (positions -71 and -284 respectively). In addition, we found a WT1 binding site in the antisense strand (pos. -112). Only a few other upstream regulatory elements were defined within and proximate to the CpG rich region. We found one binding motifs associated to Ets-2 [19], one to the c-myc purine-binding transcription factor PuF [20] and one to the early growth response gene 2 (table 2, Fig. 4).

**Figure 5:**
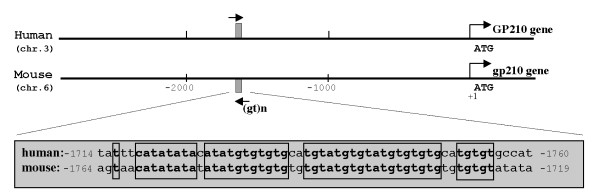
Alignment of mouse and human sequences demonstrating a conserved region about 1700 bp upstream of ATG.

A sequence of 9433 bp (c047302867. Contig1) was found in the Mouse Genome Sequencing Consortium (MGSC) database. This sequence mapped to mouse chromosome 6 and contained the first exon, part of the first intron, and 3 kb of the upstream promoter region of mouse GP210. Similar to human, a GPC island containing 644 bp and with a GC content of 74% was found starting 268 nt upstream of the start codon and extending into the gene. Pairwise ClustalW alignment of this genomic clone showed 54% homology to its human counterpart within the first 500 bp upstream of the translation start (fig. 4). In the same region as in human we found putative EGR1/WT1 binding sequences. The sequence at positions -40 to -32 matched completely with the consensus sequence. The sequences starting at -47, -76, and -283 had one, two and one mismatches, respectively. As in the human sequence, an additional putative WT1 binding site was located in the antisense strand starting at position -83, but it contained a single nucleotide insertion. An Ets-2 motif, identical to the motif starting at -500 in the human sequence, was found starting at position -390 in the mouse sequence. In addition, a 40 bp (GT)n repetitive sequence was located about 1700 bp upstream of the ATG both in the human and mouse promoter (Fig. 5). This repetitive sequence is known to exist redundantly in the genome [21], and has been reported to be a binding element for WT1 [22,23].

**Figure 6:**
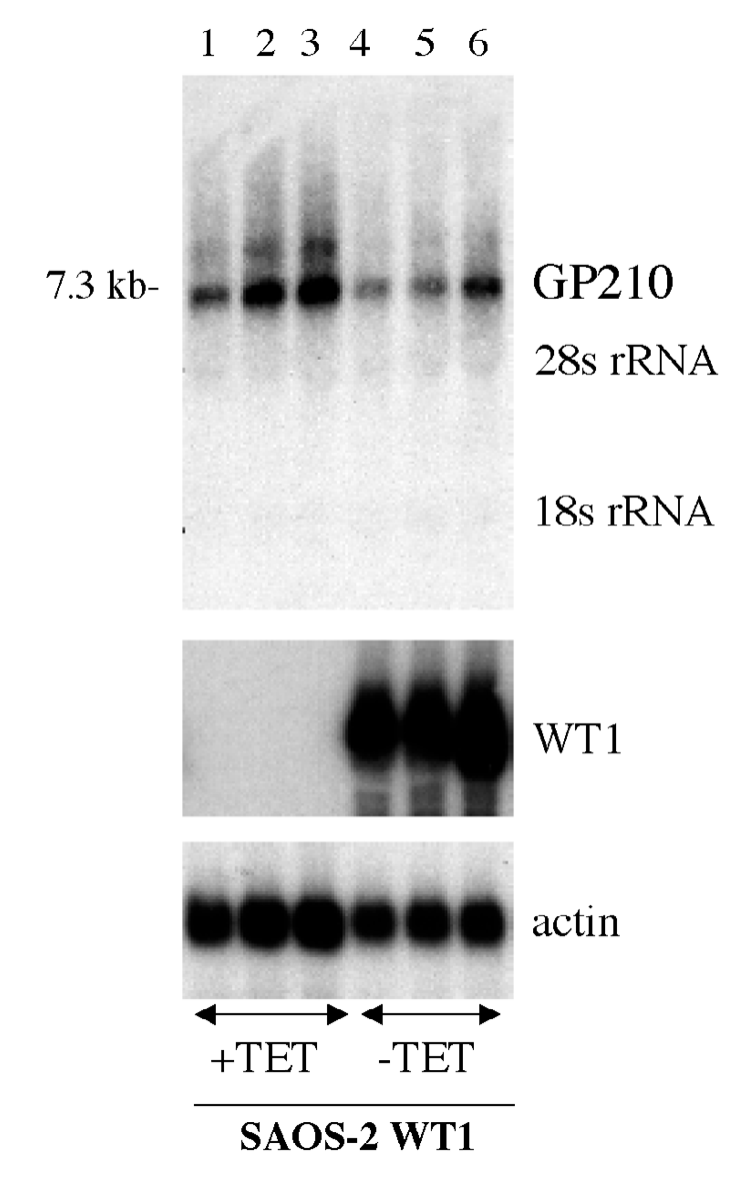
**WT1 isoform A does not regulate expression of GP210**. SAOS cells conditionally expressing WT1 isoform A were grown in the presence (Lanes 1–3) or absence (lane 4–6) of tetracycline to induce expression of WT1. Triplicate plates of cells were harvested for total RNA and subjected to sequential northern blot analysis with the GP210 probe, WT1probe and actin control.

To determine whether the putative WT1 binding sites in the GP210 promoter might correspond to functional regulation of GP210 by WT1, we used a model system for the examination of WT1 target genes in which WT1- isoform A, devoid of a 17 amino acid insert and a KTS insert in the zinc finger region, was expressed upon removal of tetracycline from the growth media. Figure 6 shows in a triplicate experiment that the induction of WT1 (Lanes 4–6) in these cells did not alter GP210 transcription. An actin control showed comparable loading and integrity of mRNA. These data suggest that GP210 is not a target of WT1.

## Discussion

The present study describes the genomic structure of the human nucleoporin GP210 gene, including its exon and intron sizes, intron/exon junctions and the 5' UTR sequence. Transcription start sites were determined experimentally by RNA ligase mediated rapid amplification of cDNA ends. Analysis of the promoter sequence identified a number of putative binding motifs for factors involved in tissue- or cell -type specific gene regulation. Strikingly, we could identify five putative Wilms tumor 1 (WT1) suppressor protein binding sites, four Sp1 biding sites, and one ETS binding site in a range of 315 bases just upstream of the translation initiation site. Some of these were conserved in the mouse promoter region.

Mouse GP210 was initially identified as an early response gene to induction of embryonic kidney tubule development [10,15], suggesting that transcription factors regulating conversion of mesenchyme to kidney tubules are involved in its activation. Transcription factors implicated in early kidney tubule development include members of the myc family, Pax-2, hox a11 and Hoxd11, lmx-1b, HNF-1a, Pod-1, and WT1 [24-29]. Except for the WT1 binding site, putative binding motifs for these factors were lacking in the promoter region of the GP210 gene. Experimentally we found that WT1 does not influence GP210 expression in human osteosarcoma cells. It is thus more likely that Sp1 and some member of the ETS transcription factor family are the positive regulators of GP210.

WT1 is a zinc finger transcription factor known to exist in different isoforms due to alternative pre-mRNA splicing. DNA binding specificity is determined by insertion or removal of three amino acids between zinc finger III and IV (referred to as WT1(+KTS) and WT1(-KTS)). The -KTS isoform have been reported to repress or activate target genes containing variations of an EGR1 related, GC-rich motif (consensus GXGXGGGXG) in their promoter [24]. Other biological activities have been suggested for the +KTS isoform [26]. Mutations in the WT1 gene has been shown in a small proportion of nephroblastomas, an embryonic kidney tumor, as well as in other tumor types, such as leukemia, mesothelioma and desmoplastic small round cell tumor. The restricted expression pattern in the mouse embryonic kidney and the failure of kidney development in WT1 null mice shows that WT1 is important for mesenchyme-to-epithelial transition, especially for early organogenesis of kidney and gonads [29]. It is thus of considerable importance to identify downstream target genes for WT1. This could include the gene for GP210, but presumably not other nucleoporins. In the only previously reported nucleoporin promoter region, of mouse nup358, several binding sites for Sp1 but none for WT1 were detected [25,30].

Sp1 is a transcription factor included in a small protein family (Sp1, Sp2, Sp3, and Sp4), whose members are binding to *cis*-elements widely distributed in different types of transcription control regions [25]. Although traditionally considered as an activator for house keeping genes, it has become increasingly clear that Sp1 can act as a cell specific regulator of gene expression. Differential expression levels of Sp1 during nephrogenesis [31] and hematopoietic development [32] have been reported. Along with specific post-translational modifications, the substantial differences in the expression patterns of Sp1 suggest that Sp1 can induce specific gene expression in embryonic tissues, including GP210 in the kidney.

We also found a putative ETS-2 binding site in the mouse and human promoter for gp/GP210. Ets-2 is a widely distributed member of the ETS family of transcription factors characterized by a unique winged helix-turn-helix domain, which specifically interacts with DNA sequences containing the purine-rich core motif, GGAA/T. Since several ETS family members binds to the same core motif it has been difficult to determine specific target genes for each member, gene targeting in mice implicates ETS-2 as an activator of metalloproteinases in placenta (MMP-3, MMP-9 and MMP-13) and a regulator of hair development [33]. Elevated ETS-2 expression can reverse ras dependent transformation in cell lines [34]. In contrast, a high expression of ETS-2 is needed to maintain the transformed state of human prostate cells [35]. These data suggests multiple roles for ETS-2 during development and cancer. Interestingly, a binding site for Pea3, a member of the ETS family, has been noted in the WT1 promoter, and Pea3 was found to transactivate the Wt1 promoter [36].

Our promoter region analyses, which identify WT1, SP1 and Ets-2 as putative transcription factors regulating GP210 expression are descriptive. GP210 expression in the developing kidney resembles that of E-cadherin, which has been show to be a bone fide WT1 target gene [24]. WT1 A isoform, which lacks a 17 amino acid insert and the KTS insert in the zinc finger region, did not influence GP210 expression in SAOS osterosarcoma cells, in a system using tetracycline-induced repression of expression. In vivo, expression of WT1 appears very early during nephrogenesis, and is downregulated when GP210 expression increases [10,15,28,29]. Based on these findings, it was difficult to predict whether WT1 is involved in the positive or negative regulation of GP210. Our data do not exclude the possibility that demonstrate that the WT1 A isoform in different setting can regulate GP210 expression. It is also possible that other isoforms of WT1 regulate GP210.

The amino acid sequence of human GP210 revealed potential sites for phosphorylation and glycosylation. The role for phosphorylation of nuclear envelope associated proteins is not well understood, but is presumed to have a function in mitotic events [37,38]. Non-membrane nucleoporins 153, 214 and 358 are phosphorylated throughout the cell cycle, but hyperphosphorylated during cell division. In contrast, GP210, was in the same study specifically phosphorylated during mitosis and one single consensus Ser^1880^-Pro^1881 ^motif could be detected as a target for cyklin-B-p34^cdc2 ^kinase and MAP kinase in vitro [39]. A comparison of the cytoplasmic domain in mouse, rat and human reveals that this serine-proline dipeptide located seven amino acids downstream of the carboxyl terminus is conserved. Whether the many putative phosphorylation sites in GP210 are actively regulated has to be experimentally determined. Restricted to the lumenal region of GP210, there are 12 potential putative acceptor residues for N-linked oligosaccharides. This is one residue less than in the rat homologue [5], but the remaining 11 seem to be located at conserved locations. The binding of GP210 to the lectin ConA suggests presence of high mannose-type oligosaccharides in mature GP210 [4], but there are no reports on functional aspects of this posttranslational modification.

## Materials and methods

### Wilms tumor and HeLa cell cultures

WCCS-1 Wilms tumor cells [40] and HeLa cells were cultured in Dulbecco's modified medium containing 10% heat- inactivated Fetal Calf Serum (FCS) and in the presence of 1% HEPES. RNA from WCCS-1 and HeLa cells was isolated from 5 × 10^7 ^cells using the RNAeasy midi kit (Qiagen) following the recommendations of the manufacturer, including a DNAse treatment step to avoid chromosomal DNA contaminations. Northern blotting of total RNA from cells was performed as described [41]. To visualize 18 and 28s rRNA, a control RNA filter lane were immersed in 5% acetic acid followed by colorization in 0,5 MNaAc (pH 5,2), 0,4% methyleneblue for 15 min. cDNA probes used: a 935 bp PCR generated GP210 probe (nt 4286–5220), a 1.8 kb human β-Actin control probe (Clontech) and a 1023 bp PCR generated human WT1 probe covering the 3' end of the ORF and 523 bp of the 3' untranslated region thereby hybridizing to all known splice variants [42]. Probes were labelled with [α^32^P] dCTP by random priming using Megaprime DNA labeling system kit (Pharmacia). Filters were hybridized in 20 mM Na_2_HPO_4 _(pH 7.2), 7% SDS at 65°C for 18 hours. After washing in 20 mM Na_2_HPO_4 _(pH 7.2), 5% SDS at 65°C for 2 × 60 min followed by 2 × 60 min in 20 mM Na_2_HPO_4 _(pH 7.2), 1% SDS at 65°C the filters were exposed to Hyperfilm-MP films (Amersham) for 5 days at -70°C in the presence of intensifying screens. Band intensities were quantified using a PhosphoImager 400S (Molecular Dynamics, Sunnyvale, CA).

### Tetracycline-regulated expression of WT1 in human osteosarcoma cells

WT1-A SAOS cells were constructed from a cell line harboring the tetracycline-repressor-VP16 fusion protein [43], transfecting the parental cell with a construct harboring WT1 isoform A (-17amino acids, -KTS) linked 3' to the CMV minimal promoter and tetracycline operators [44]. Conditional expression of WT1-A was demonstrated by immunoblotting. WT1-A-SAOS cells were maintained in Dulbecco's modified Eagle's medium supplemented with 10% fetal calf serum, 1∞ penicillin/streptomycin, 0.3 mg/mL L-glutamine and 0.5 mg/mL G418. All cells were cultured at 37°C in a 5% CO_2 _atmosphere. For the induction, cells (at 70% confluence) were washed twice with PBS and refed with fresh media in the absence or presence of 1 μg/mL of Tetracycline. After 18 hours, the cells were washed twice with PBS and total RNA was isolated using TRIzol reagent (Invitrogen, Carlsbad, CA) according to the manufacture's instructions. 4 μg of the RNA were resolved on formaldehyde-containing agarose gels and transferred to Nytran membranes (Schleicher and Schuell, Keene, NH). Hybridization was performed in ULTRAhyb buffer (Ambion, Austin, TX) at 42°C. Briefly, filters were prehybridized in ULTRAhyb buffer for 6 hours followed by an overnight hybridization at 42°C with the 935 bp hGP210cDNA probe. A WT1 cDNA probe (exons 5 to 10) and a cDNA probe for actin were used as controls. Membranes were exposed to BIOMAX MS films (Kodak, Rochester, NY) at -80°C in the presence of intensifying screens. Probes were stripped by boiling the membranes in 0.1% sodium dodecyl sulfate/ standard saline citrate solution for 10 minutes.

### Oligo-capping

To determine transcription start, the RNA Ligase Mediated Rapid Amplification of cDNA Ends (RML-RACE) kit (Ambion) was used according to the instructions of the manufacturer's. Briefly 5 μg of Hela cell RNA or 2–5 μg of poly-A+ RNA from partially differentiated human G6 satellite cells [45] was treated with calf intestinal phosphatase (CIP) at 37°C for 60 min. RNA from G6 cells was kindly provided by Donald Gullberg at ICM, Uppsala University, Sweden. The mixture was phenol:chloroform (1:1) extracted followed by ethanol precipitation. The RNA was subsequently incubated with Tobacco Acid Phosphatase (TAP) at 37°C for 60 min. A 45 nt adapter RNA oligonucleotides (5'GCUGAUGGCGAUGAAUGAACACUGCGUUUGCUGGCUUUGAUGAAA3') was ligated to the CIP/TAP treated 5' RNA end using T4 ligase. cDNA was generated using random decamers and MMLV reverse transcriptase at 42°C for one hour. The nested PCR were performed using the advantaq 2 polymerase system (Clontech) and the following primers: in the first PCR reaction the outer adapter (5'GCTGATGGCGATGAATGAACACTG3') was combined with the gene specific outer primer (5'CAGCAGCACTTTGGGGATGTTGAG3'), in the second PCR the inner adapter primer (5'CGCGGATCCGAACACTGCGTTTGCTGGCTTTGATG-3') was used in combination with the gene specific inner primer (5'CCCGCCGCCAACAGCACCGACAGC3'). The conditions for both PCR reactions were as follows: 94°C for one min (hot start) followed by 95°C for 20 s, 68°C for 60 s, repeated 35 cycles. After the final cycle, the reactions were extended for an additional 5 min at 68°C. PCR products were analysed on a 2% agarose gel, ligated into the pCRII vector (Invitrogen) and sequenced using M13 primers.

### Sequencing

Sequencing was performed using ABI PRISM and the Dye Terminator cycle sequencing kit according to the manufacturer's directions (Perkin Elmer) and analysed by an automated ABI-310 fluorescent-dy sequencer (Applied Biosystems).

### Bioinformatics and sequence analyses

Exon-intron boundary predictions were done manually and using the Genscan web server at MIT . Open reading frame finding and all sequence analyses were done using the MacVector 6.5.3 sequence analysing software (Oxford Molecular Group). The BLAST (Basic Local Alignment Search Tool) server of the National Center of Biotechnology Information (NCBI)  and The Mouse Genome Sequencing Consortium (MGSC) database  was used for sequence alignments. Signal peptide prediction was done using the SignalP v1.1 at WWW Prediction Server (Center for Biological Sequence Analysis, and membrane topology predictions at the HMMTOP server using the TopPred2 program [46]. Transcription factor mapping in the 5' untranslated region of hGP210 was analysed in TESS  and Matinspector [47] search programs. Additional amino acid sequence patterns and domains were analysed using the PROSITE database . Prediction of GPC islands and detection of repeats in sequences analysed were done with the GRAIL program [48].

## Supplementary Material

Additional File 1 Table 1. Organization of the human GP210 gene including exon/intron sizes, splice acceptor consensus sequences and intron phases. Click here for file

Additional File 2 Table 2. Predicted *cis*-acting elements of the human GP210 promoter. The start sites of the elements are indicated as 5' end of consensus sequence and relative to translation start as +1. The compilation was made using the TESS and MatInspector analyze programs. Results are restricted to human species and perfect match except for four WT1 binding sites indicated as a boxed nucleotide. Some abbreviations: EGR2, Early growth response gene 2; WT1, Wilms' tumor zinc finger protein 1; PuF, c-myc purine-binding transcription factor; IL-6. RE-BP, IL-6 Response element-Binding protein; NF-1, Nuclear factor 1; Ets-2, proto-oncoprotein; USF2, upstream stimulating factor. Click here for file
