# Supplementary figures and images for: Despite WT1 binding sites in the promoter region of human and mouse nucleoporin glycoprotein 210, WT1 does not influence expression of GP210
Source: J Negat Results Biomed. 2004 Dec 21;3:7. doi: 10.1186/1477-5751-3-7 (PMC544869; doi:10.1186/1477-5751-3-7)

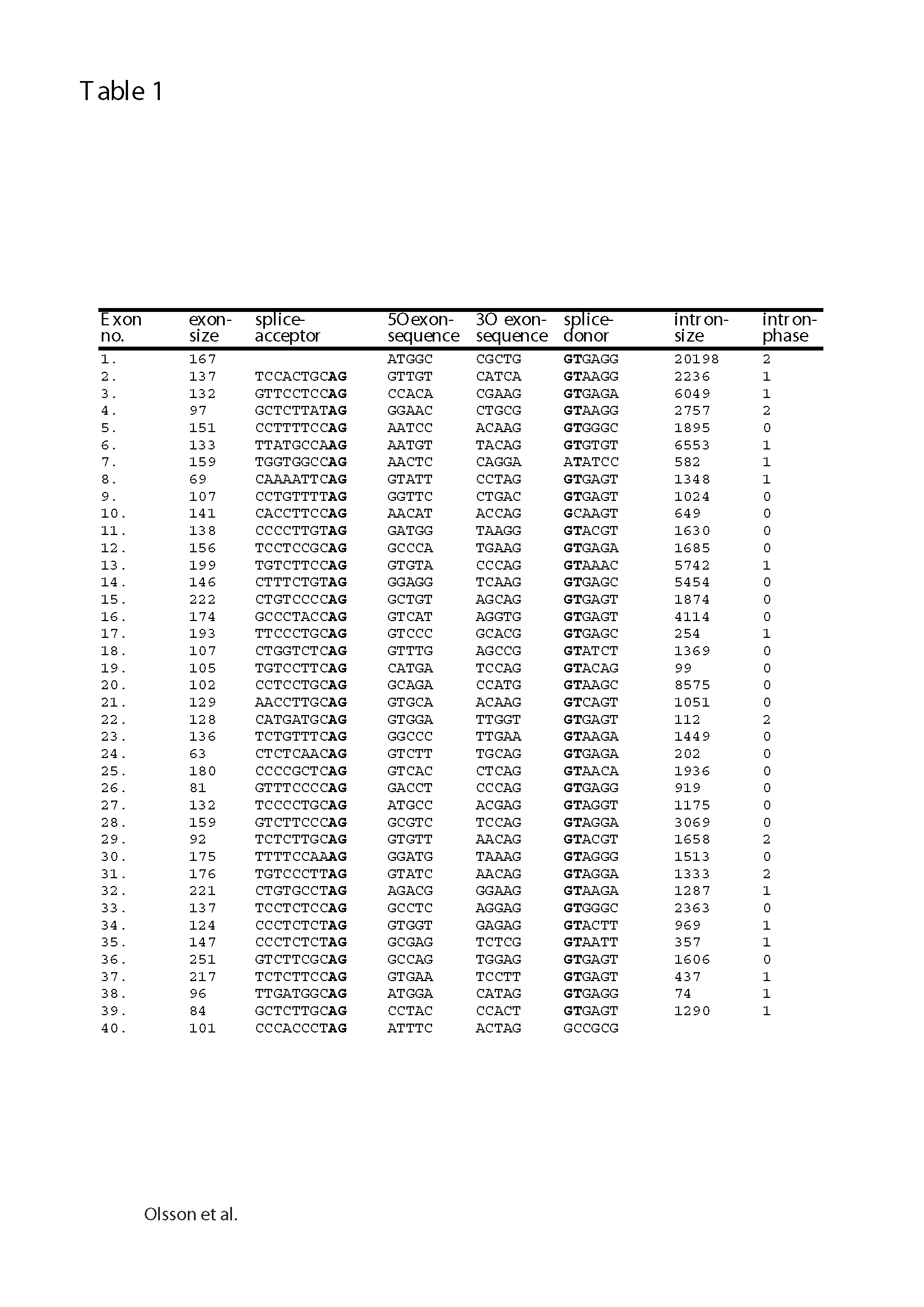

Supplement: Additional File 1 — Table 1. Organization of the human GP210 gene including exon/intron sizes, splice acceptor consensus sequences and intron phases. [file 1477-5751-3-7-S1.TIFF]

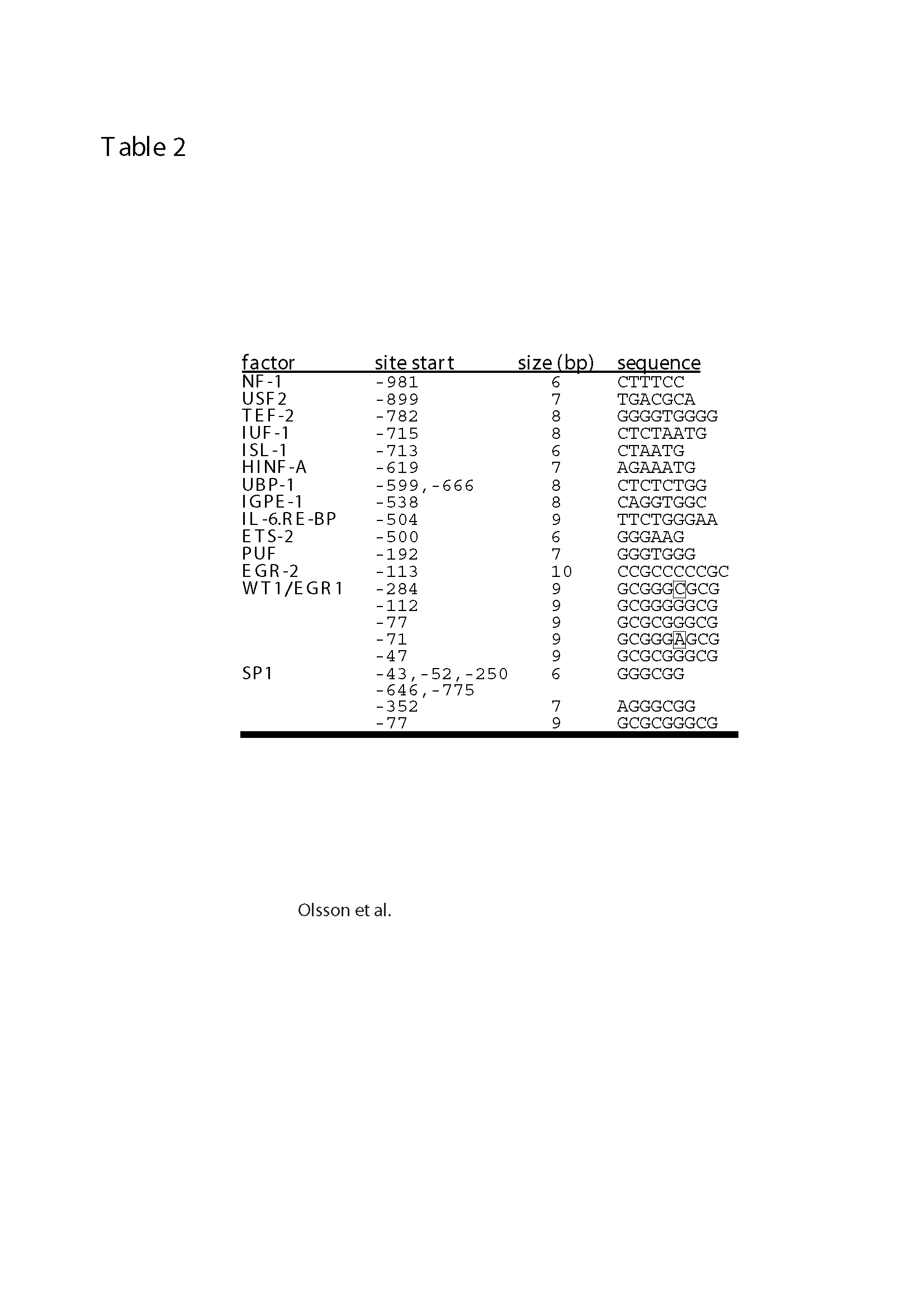

Supplement: Additional File 2 — Table 2. Predicted cis-acting elements of the human GP210 promoter. The start sites of the elements are indicated as 5' end of consensus sequence and relative to translation start as +1. The compilation was made using the TESS and MatInspector analyze programs. Results are restricted to human species and perfect match except for four WT1 binding sites indicated as a boxed nucleotide. Some abbreviations: EGR2, Early growth response gene 2; WT1, Wilms' tumor zinc finger protein 1; PuF, c-myc purine-binding transcription factor; IL-6. RE-BP, IL-6 Response element-Binding protein; NF-1, Nuclear factor 1; Ets-2, proto-oncoprotein; USF2, upstream stimulating factor. [file 1477-5751-3-7-S2.TIFF]
